# Lipid have a direct effect on multiple myeloma: a Mendelian randomization study

**DOI:** 10.3389/fonc.2024.1404744

**Published:** 2024-06-11

**Authors:** Yingbin Zhong, Yanhao Li, Weipeng Sun, Mingfeng Xiao

**Affiliations:** ^1^ Guangzhou University of Chinese Medicine, Guangzhou, China; ^2^ The First Affiliated Hospital of Guangzhou University of Chinese Medicine, Guangzhou, China; ^3^ Guangdong Clinical Research Academy of Chinese Medicine, Guangzhou, China; ^4^ First Clinical Medical College, Guangzhou University of Chinese Medicine, Guangzhou, China; ^5^ College of Traditional Chinese Medicine, Jinan University, Guangzhou, China

**Keywords:** multiple myeloma, liposome, single nucleotide polymorphisms, Mendelian randomization, genome‐wide association study, causal relationship

## Abstract

**Background:**

Multiple myeloma (MM), a malignant disease of plasma cells originating in the bone marrow, is influenced significantly by genetic factors. Although plasma lipid have been linked to MM, the nature of their potential causal relationship remains to be elucidated. This study aims to explore this relationship using Mendelian randomization (MR) analysis.

**Methods:**

Liposome-associated genetic instrumental variables (IVs) were identified from plasma lipidomics data of 7,174 Finnish individuals within a Genome-Wide Association Study (GWAS) pooled database. A MM pooled dataset was sourced from a GWAS meta-analysis encompassing 150,797 individuals, including 598 MM patients and 218,194 controls. These IVs underwent MR analysis, adhering to strict criteria for correlation, independence, and the exclusion of confounders. The inverse variance weighted (IVW) method, MR-Egger method, weighted median (WM) method, and simple median were utilized for MR analysis assessment, alongside Cochran’s Q test, MR-Egger intercept, MR-Pleiotropy Residual Sum and Outlier (MR-RESSO) method, and leave-one-out analysis for evaluating heterogeneity, multiplicity, and instrumental bias.

**Results:**

The study identified 88 significant, independent single nucleotide polymorphisms (SNPs) as IVs for MR analysis, each with an F-statistic value above 10, indicating robustness against weak instrument bias. IVW analysis revealed associations between six plasma liposome components and MM risk (p < 0.05). Phosphatidylinositol (16:0_18:1) serum levels (odds ratio [OR] = 1.769, 95% confidence interval [CI]: 1.132-2.763, p = 0.012) and triacylglycerol (56:4) levels (p = 0.026, OR = 1.417, 95% CI: 1.042-1.926) were positively correlated with the risk of multiple myeloma development. Phosphatidylethanolamine (18:0_20:4) (p = 0.004, 95% CI: 0.621-0.916, OR = 0.754), phosphatidylcholine (18:2_20:4) (p = 0.004, OR = 0.680, 95% CI: 0.519-0.889), sterol ester (27:1/18:3) levels (p = 0.013, OR = 0.677, 95% CI: 0.498-0.922), and phosphatidylcholine (O-18:2_20:4) levels (OR = 0.710, 95% CI: 0.517-0.913, p = 0.033) were negatively associated with the risk of developing multiple myeloma. The Cochran’s Q test did not detect statistical method heterogeneity, nor did the MR-RESSO test or the MR-Egger intercept detect horizontal pleiotropy; leave-one-out analyses confirmed the absence of bias from individual SNPs

**Conclusions:**

Our findings suggest a complex relationship between plasma liposome components and MM risk. Elevated serum levels of triacylglycerol and phosphatidylinositol are positively associated with MM risk, while certain phospholipids and sterol esters offer a protective effect. This study provides valuable insights into the clinical relevance of lipid in the pathology of multiple myeloma.

## 1 Introduction

Multiple Myeloma (MM) is a malignancy of plasma cells originating in the bone marrow, characterized by the abnormal proliferation of monoclonal plasma cells and associated hematological and skeletal complications (1). Despite its rarity, accounting for 1% of all cancers, MM ranks as the second most prevalent hematological malignancy following lymphoma. Current estimates suggest that nearly 230,000 individuals globally will be diagnosed with MM over the next five years (2). The disease’s hallmark includes the overproduction of immunoglobulins, leading to a spectrum of complications such as anemia, bone degradation, renal insufficiency, and hypercalcemia (3). MM’s pathogenesis is attributed to a combination of genetic and environmental factors, resulting in the malignant transformation of plasma cells and excessive monoclonal immunoglobulin production (4). Recent advancements in treatment, including proteasome inhibitors, immunomodulatory drugs (IMiDs), monoclonal antibodies, and histone deacetylase inhibitors, have significantly improved management outcomes. However, the relapse and mortality rates remain high, underscoring the need for continued development of novel therapeutic strategies to enhance long-term survival in MM patients (5, 6).

Recent investigations suggest a significant role for specific human serum lipid components in MM pathogenesis. Notably, acidic sphingomyelinase activity is markedly decreased in the blood of patients with MM (7), and sphingolipids have been implicated in the disease’s etiology (8). Additionally, serum triglyceride levels may correlate with MM severity (9). Despite these insights, challenges such as limited sample sizes, insufficient clinical evidence, study biases, and confounding factors have resulted in inconsistent conclusions regarding the causal relationship between liposome components and MM.

Mendelian randomization (MR) employs genetic variations as instrumental variables to ascertain the impact of environmental or exposure factors on disease outcomes. This method capitalizes on the principle that alleles are allocated to individuals before any exposure, and their distribution is largely independent of potential confounding factors encountered later in life, such as environmental influences, socio-economic status, and personal behaviors (10). Consequently, gene-disease associations deduced through MR are not affected by these common confounders, enabling the methodology to circumvent issues related to confounding and reverse causality effectively. Therefore, the aim of our study is to use Mendelian randomization to determine whether there is a genetic causal relationship between lipid and MM.

## 2 Materials and methods

### 2.1 Methods

**Figure 1:**
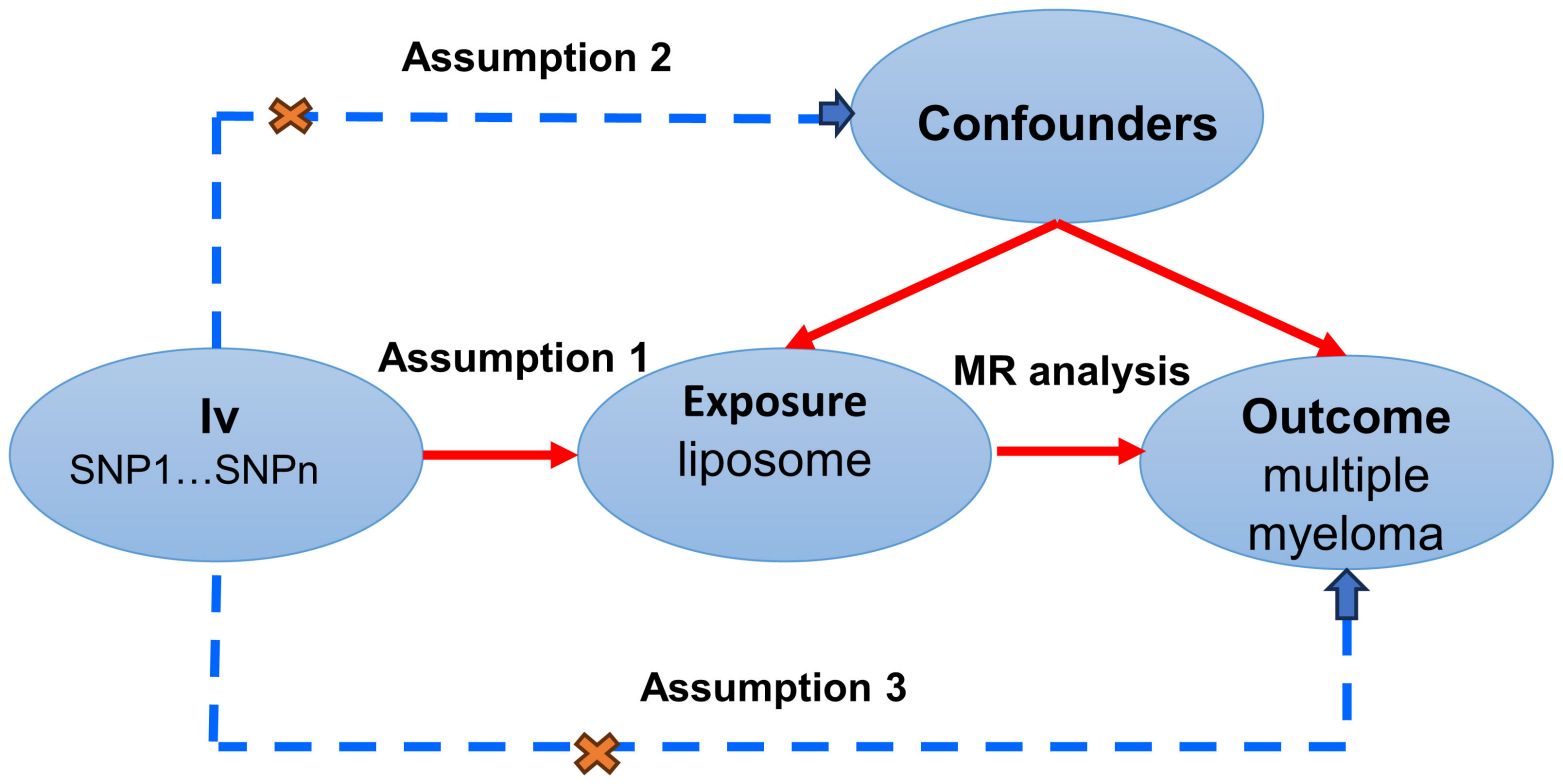
A comprehensive examination of the three assumptions of MR study.

MR studies were conducted to elucidate the causal dynamics between plasma lipid and multiple myeloma, with a detailed schematic of the study design depicted in **Figure 1**. For the MR approach to yield valid conclusions, it must satisfy three critical conditions: (A) The selected genetic variant, serving as the instrumental variable (IV), must exhibit a robust association with plasma lipid, ensuring its validity as a proxy for exposure. (B) The genetic instrument should maintain independence from the multiple myeloma outcome and not be confounded by other variables, affirming its specificity and eliminating potential bias. (C) The relationship between the genetic variant and multiple myeloma must be mediated exclusively through plasma lipid, with no alternative pathways influencing the outcome, thus ensuring the integrity of the causal inference.

#### 2.1.1 Liposome data

The study leveraged comprehensive summary statistics from the research conducted by Linda Ottensmann et al. (11), which explored the genetic influences on human plasma lipid. This foundational data, drawn from the GeneRISK cohort encompassing 7,174 Finnish individuals, underwent rigorous quality control before the initial genome-wide discovery analysis of single-nucleotide polymorphisms (SNPs) in this population. The cohort analysis identified 179 lipids across 13 lipid classes, encapsulating the primary lipid categories: glycerolipids, glycerophospholipids, sphingolipids, and sterols. The summary data from the plasma lipid genome-wide association study (GWAS) is accessible in the GWAS catalogue (https://www.ebi.ac.uk/gwas/), under the accession numbers GCST90277238 to GCST90277416. For detailed GWAS findings, refer to **Supplementary Table S1.**

#### 2.1.2 Data on MM and confounders

Summary data for single nucleotide polymorphisms (SNPs) linked to multiple myeloma were sourced from the IEU Open GWAS database (https://gwas.mrcieu.ac.uk/), identified by the dataset number finn-b-CD2_MULTIPLE_MYELOMA_PLASMA_CELL. This data was compiled from a comprehensive GWAS meta-analysis involving 150,797 individuals, including 598 patients diagnosed with multiple myeloma and 218,194 controls. Diagnosis criteria for multiple myeloma patients adhered to the International Classification of Diseases (ICD) versions 8, 9, and 10, as specified by the Finnish Genetic Alliance. The statistical data utilized in this study are publicly accessible, negating the need for ethical approval. For further details on the outcome data, refer to **Supplementary Table S2.**

### 2.2 Instrumental variable selection

In our initial analyses, we identified SNPs exhibiting p-values beneath the genome-wide significance threshold (5 × 10^-6) as IVs to enhance the comprehensiveness of our results and increase sensitivity. To mitigate the influence of correlated SNPs, all IVs underwent linkage disequilibrium (LD) clustering with parameters set at r^2 = 0.001 and a maximum distance of 10,000 kb. Furthermore, to detect any potential pleiotropic effects, we employed Phenoscanner (http://www.phenoscanner.medschl.cam.ac.uk/) to exclude SNPs linked to the outcome, as detailed in **Supplementary Table S3.**

The F-statistic formula [R^2(N-2)/(1-R^2)], where R^2 denotes the proportion of variance explained by the genetic instrument and N represents the effective sample size of the GWAS, was utilized to evaluate the strength of each IV. Only SNPs with F-statistic values exceeding 10 were included in subsequent MR analyses to ensure reliable estimates of genetic variance.

### 2.3 Mendelian randomization analysis

To investigate the causal linkage between 179 lipid and multiple myeloma, we executed two-sample MR analyses employing various models and tests. These included sum-weighted models, inverse variance weighted (IVW) tests, with the IVW method serving as the primary test in scenarios devoid of horizontal pleiotropy or when such pleiotropy was neutralized, thereby providing an unbiased estimation of the causal effects between exposures and outcomes.

For sensitivity analyses, acknowledging the trade-off with statistical power, we applied weighted median and MR-Egger regression tests, which accommodate differing hypotheses. The weighted median test permits up to 50% of the SNPs to be invalid instruments or exhibit pleiotropy. Instrument heterogeneity was evaluated using Cochran’s Q test. To address pleiotropy and outliers, we utilized MR polytropic residuals (MR-pleiotropy) and MR-PRESSO, setting a significance threshold of p < 0.05 to determine statistical significance and infer potential causal relationships.

**Figure 2:**
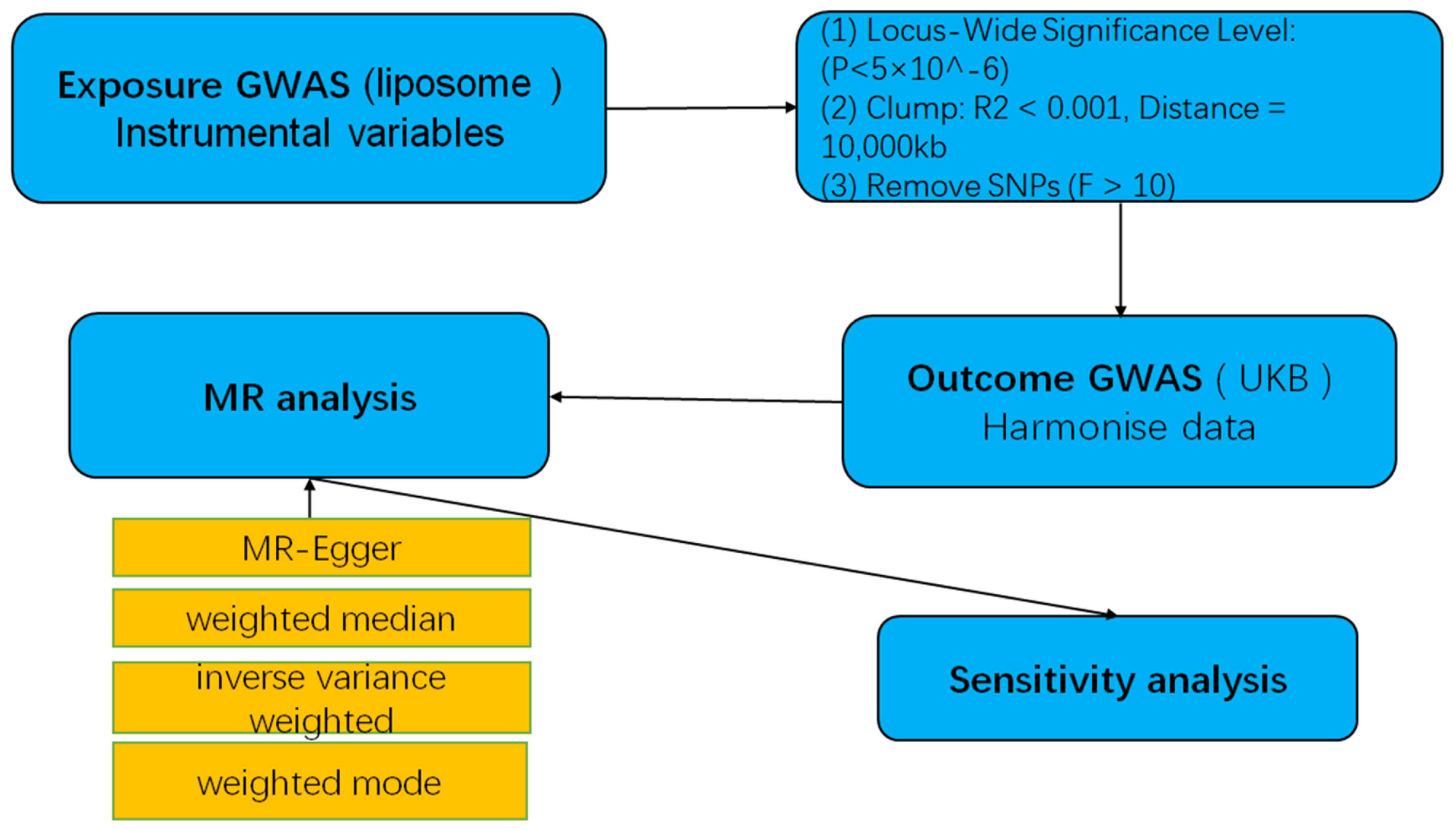
A flow-chart of MR study to explore the causal relationship between lipid and multiple myeloma.

Statistical power was calculated using the mRnd efficacy calculator (http://cnsgenomics.com/shiny/mRnd/), and results are detailed in the corresponding table. All statistical analyses were conducted in R software version 4.2.3, utilizing the TwoSampleMR and MRPRESSO software packages for MR analysis. A graphical representation of the MR research methodology is illustrated in **Figure 2**.

### 2.4 Reverse Mendelian randomization

The data sources utilized for the reverse Mendelian randomization analysis were consistent with those employed for the forward Mendelian randomization. MM served as the exposure, with SNPs closely associated with multiple myeloma selected as the instrumental variables for the exposure (p < 5 × 10^-6^). Similar to the forward Mendelian randomization approach, instrumental variables characterized by chain imbalance and F less than 10 were excluded from the analysis. The six significant plasma lipid identified in the forward Mendelian randomization analysis were chosen as the outcome variables. Subsequently, a two-sample Mendelian randomization analysis was conducted to ascertain the presence of reverse causality between MM and these significant lipid.

## 3 Results

### 3.1 Selection of IVs

In our study, we identified 88 significant and independent SNPs as IVs for MR analysis. These IVs demonstrated no correlation with the outcome of interest, multiple myeloma, ensuring the validity of our instrumental approach. The range of F-statistic values, spanning from 20.81 to 1946.15, underscored the robustness of our selected instruments by effectively mitigating the risk of weak instrumental variable bias. For detailed insights into the plasma liposome-associated SNPs, including β-values, standard errors, effector alleles, and other allele data, refer to **Supplementary Table S3.**

### 3.2 MR analysis

**Figure 3:**
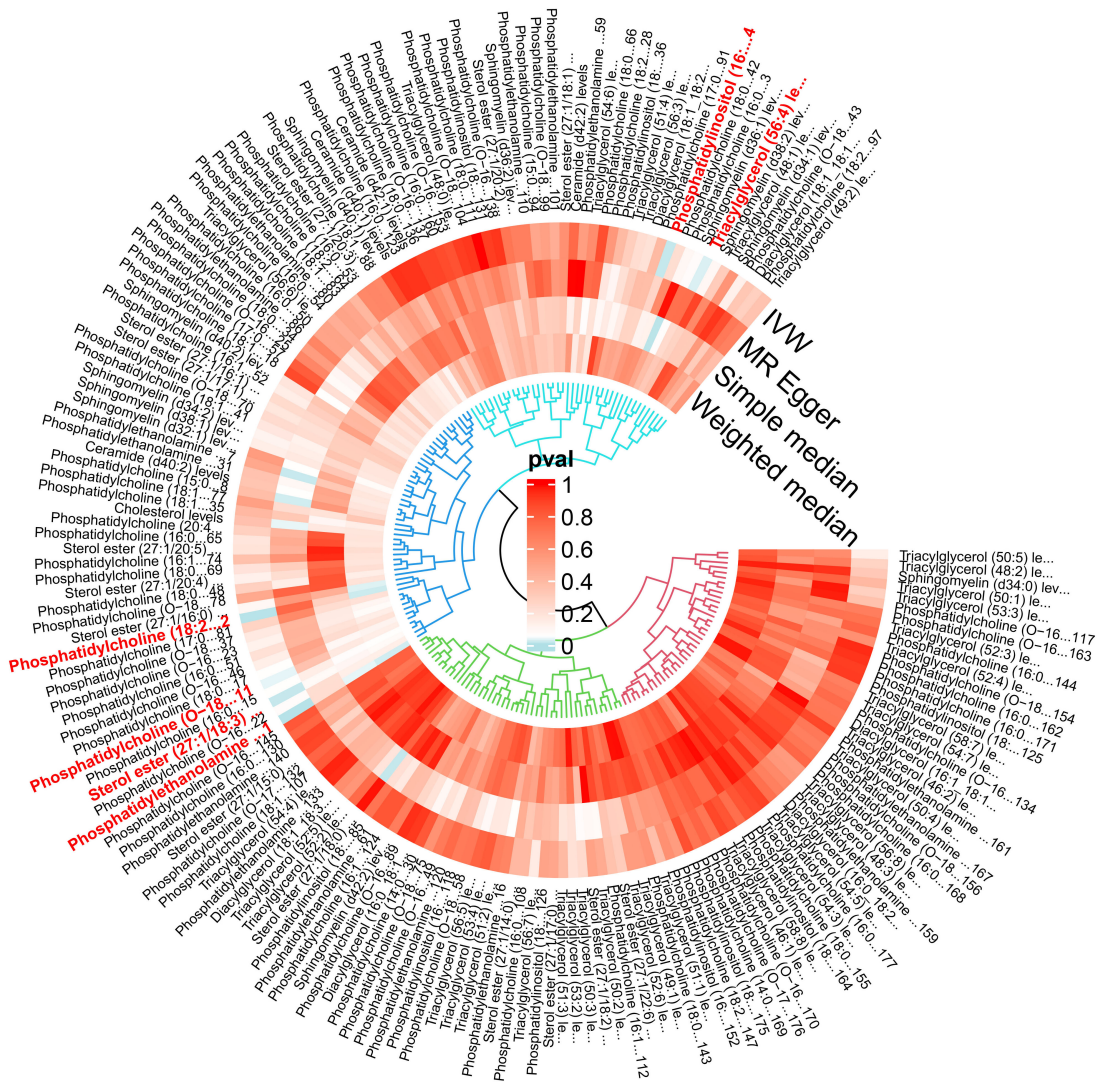
The effect of various lipid on multiple myeloma.

The IVW analysis identified a significant association between six plasma liposome components and the risk of multiple myeloma (P < 0.05), namely Phosphatidylethanolamine (18:0_20:4), Phosphatidylcholine (18:2_20:4), Sterol ester (27.1/18:3), Phosphatidylcholine (O-18:2_20:4), Phosphatidylinositol (16:0_18:1), and Triacylglycerol (56:4). These findings are visually presented in **Figure 3.**

In our study, the serum level of phosphatidylethanolamine (18:0_20:4) was negatively associated with the risk of multiple myeloma (p = 0.004, 95% confidence interval [CI]: 0.621-0.916, odds ratio [OR] = 0.754), indicating a 24.6% lower chance of developing MM with elevated levels of phosphatidylethanolamine (18:0_20:4). Phosphatidylcholine (18:2_20:4) serum levels were negatively associated with the risk of developing multiple myeloma (p = 0.004, OR = 0.680, 95% CI: 0.519-0.889), indicating a 32% lower chance of developing MM with elevated levels of phosphatidylcholine (18:2_20:4). Sterol ester (27:1/18:3) levels (p = 0.013, OR = 0.677, 95% CI: 0.498-0.922) were negatively associated with the risk of developing multiple myeloma, indicating a 32.3% lower chance of developing MM with elevated levels of sterol ester (27:1/18:3). Phosphatidylcholine (O-18:2_20:4) levels (OR = 0.710, 95% CI: 0.517-0.913, p = 0.033) were negatively associated with the risk of multiple myeloma, indicating a 29% lower chance of developing MM with elevated levels of phosphatidylcholine (O-18:2_20:4). The results indicate that elevated levels of phosphatidylethanolamine (18:0_20:4), phosphatidylcholine (18:2_20:4), sterol ester (27:1/18:3), and phosphatidylcholine (O-18:2_20:4) are associated with a reduced risk of developing multiple myeloma.

**Figure 4:**
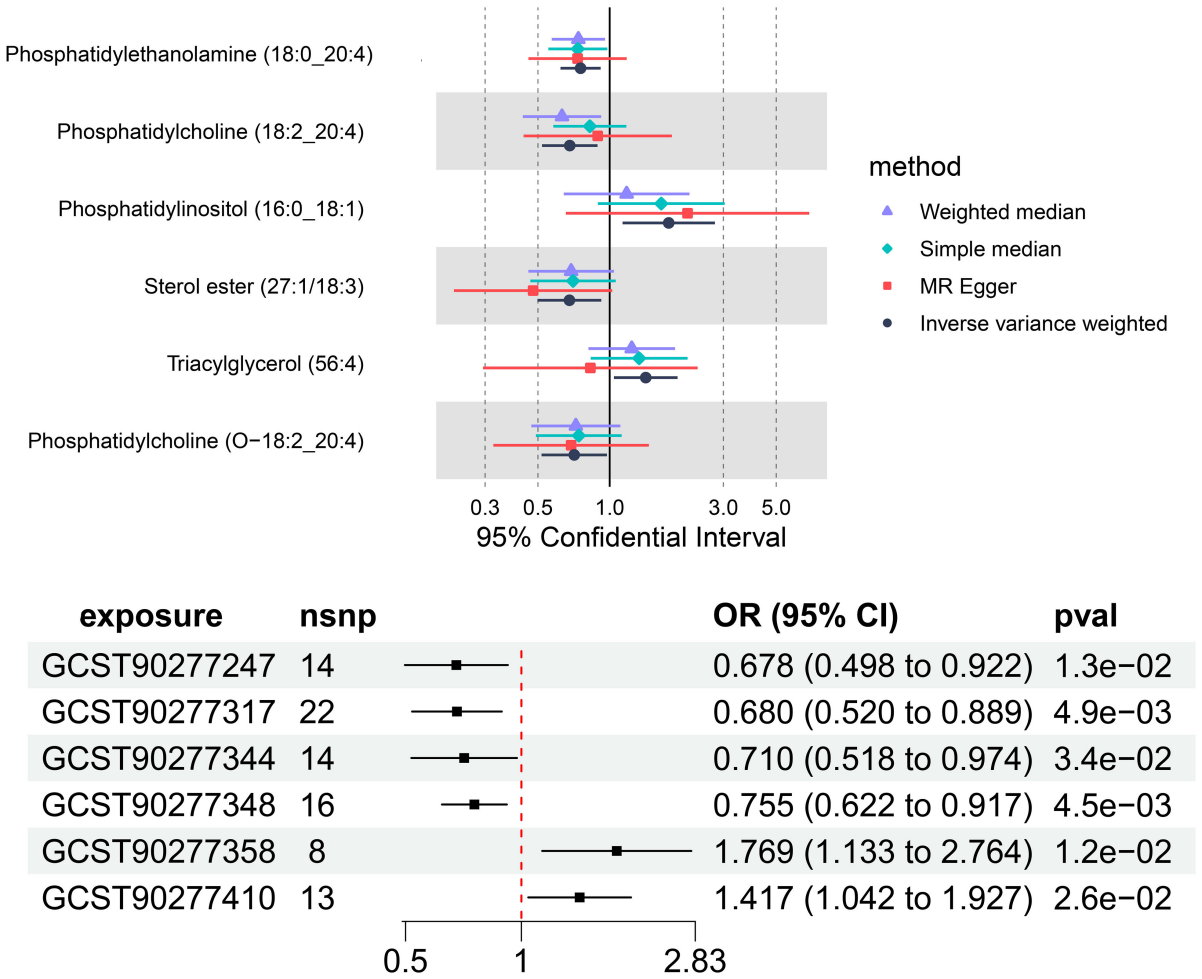
The causal effect of six plasma lipid on MM risk; GCST90277317: Phosphatidylcholine (18:2_20:4) levels; GCST90277247: Sterol ester (27:1/18:3) levels; GCST90277344: Phosphatidylcholine (O-18:2_20:4); GCST90277348: Phosphatidylethanolamine (18:0_20:4); GCST90277358: Phosphatidylinositol (16:0_18:1) levels; GCST90277410: Triacylglycerol (56:4) levels.

Our findings demonstrate that elevated serum levels of Phosphatidylinositol (16:0_18:1) (OR = 1.769, 95% CI: 1.132–2.763, p = 0.012) and Triacylglycerol (56:4) (OR = 1.417, 95% CI: 1.042–1.926, p = 0.026) are positively associated with an increased risk of multiple myeloma, indicated a 76.9% greater chance of occurring MM with elevated levels of Phosphatidylinositol (16:0_18:1) and a 41.7% greater chance of Triacylglycerol (56:4). This indicates that higher concentrations of these lipids correlate with a greater relative risk for the disease. The intricate relationship between various liposome components and multiple myeloma risk is visually represented in the deep forest plot in **Figure 4.**

### 3.3 Heterogeneity test and pleiotropy test

**Figure 5:**
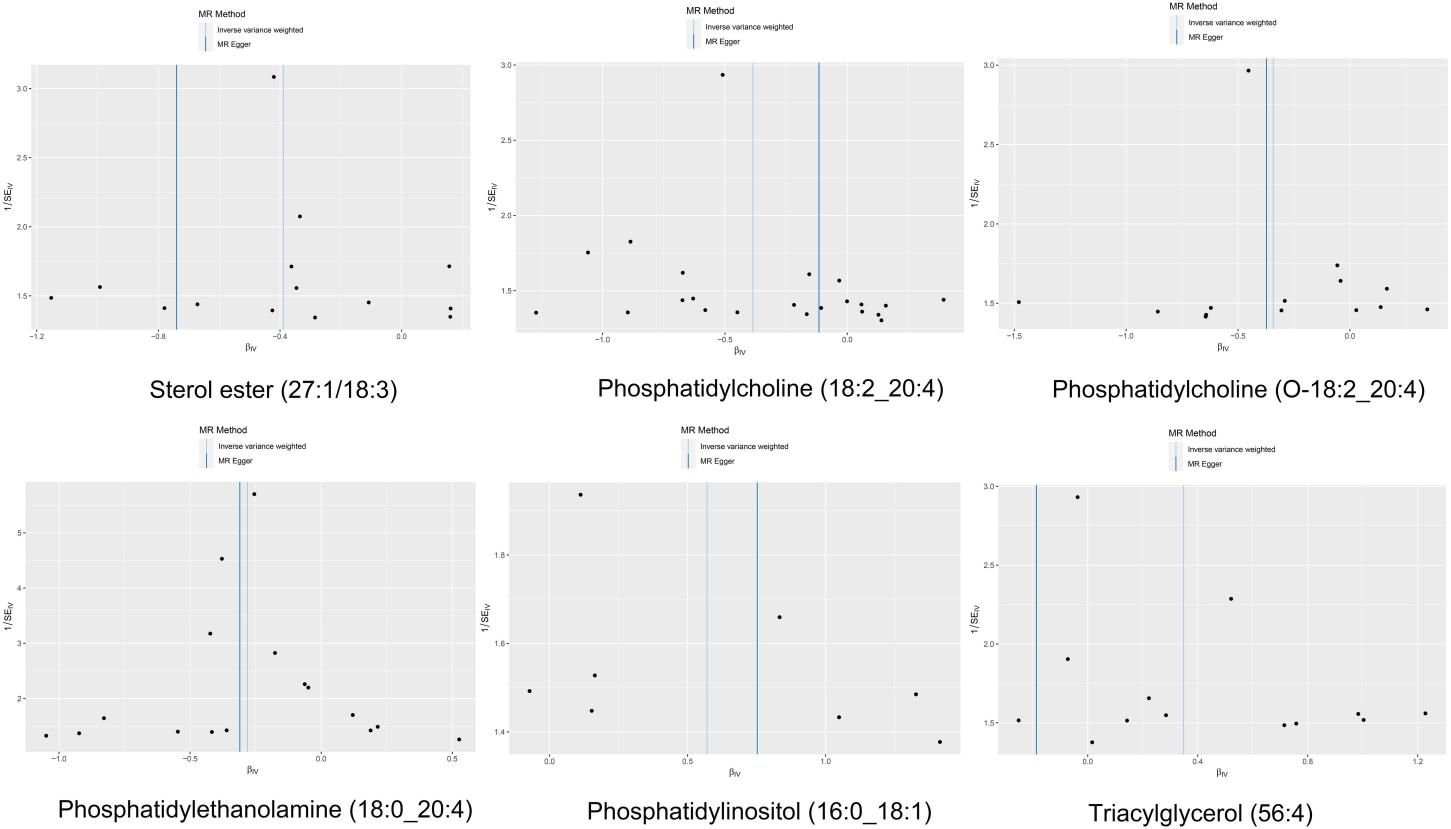
The funnel plot of the causal effect of six plasma lipid on MM risk. It’s almost symmetrical on both sides, which indicated no significant heterogeneity between the effect estimates of the IVs in both the IVW method and MR-Egger method.

**Figure 6:**
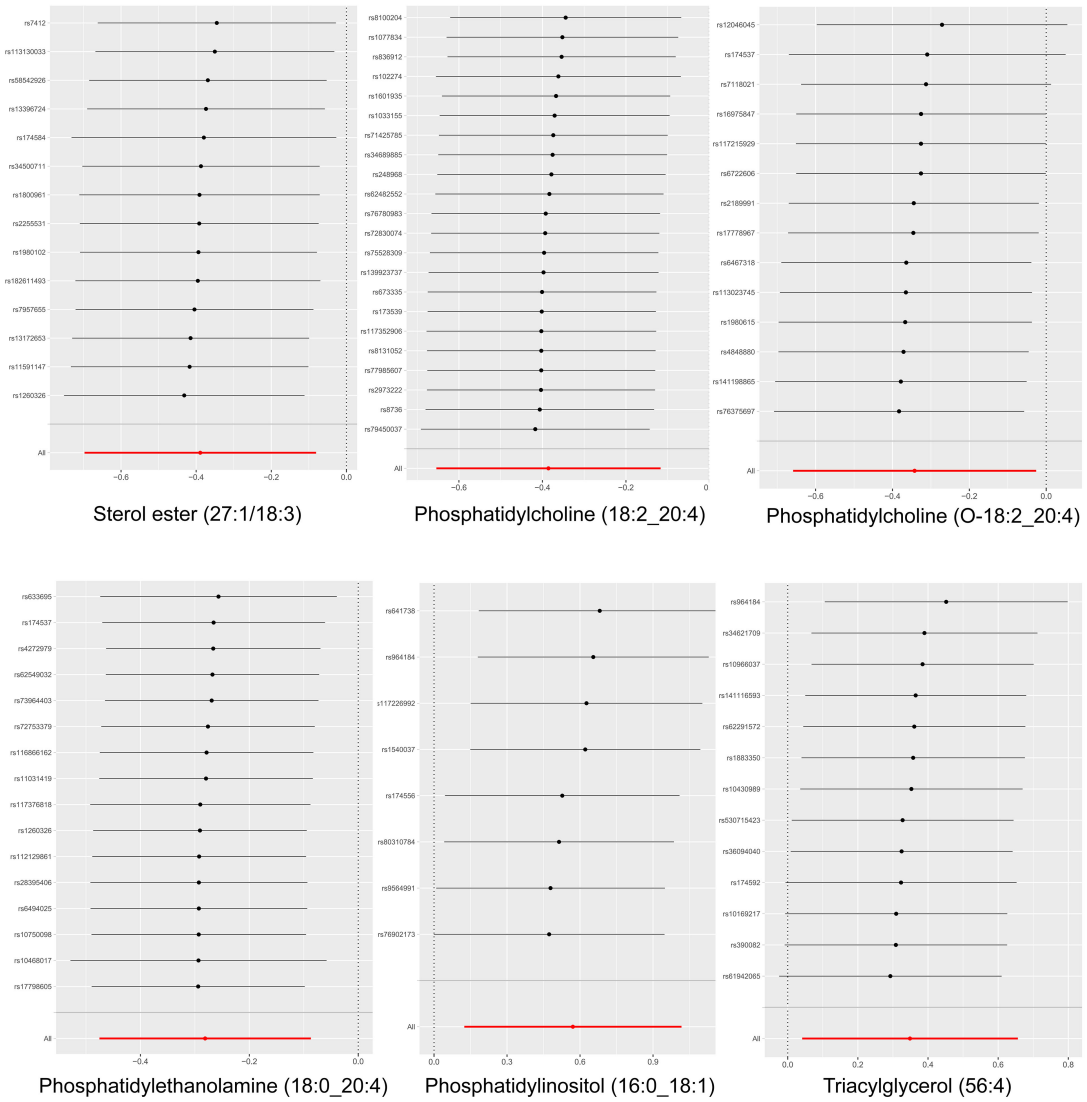
Leave-one-out analysis of the causal association between six lipid and MM. The exclusion of individual SNPs did not result in substantial differences in the combined effect estimates between the remaining SNPs and the overall results.

Cochran’s Q test revealed no significant heterogeneity between IVs effect estimates derived from the IVW method and the MR-Egger method, as illustrated in **Figure 5**. Further exploration of pleiotropy through MR pleiotropy and MR-PRESSO_Global analyses did not identify any outliers, indicating an absence of significant pleiotropy in our study findings. Additionally, MR-Egger regression analyses corroborated that pleiotropy did not influence the MR results. The integrity of our findings was further supported by sensitivity analyses, which demonstrated minimal or no impact of pleiotropy, as detailed in **Table 1**. The findings from the ‘leave-one-out’ analyses demonstrate that, upon exclusion of any individual SNP, the outcomes for the remaining SNPs consistently fall on the same side of the null line, as illustrated in **Figure 6**. This observation suggests that each SNP was included to robustly establish the significance of the causal relationship. The exclusion of any individual SNP does not materially impact the overall findings, thereby reinforcing the robustness of the MR results in this study.

**Table 1:** Results of multiplicity and sensitivity analyses of six lipid.

Description Lipid	MR-Egger		MR-heterogeneity		MR-pleiotropy		MR-PRESSO_Global		Power
	OR (95%Cl)	p-value	Cochran’s Q	p-value	intercept	p-value	RSS	p-value	
Phosphatidylethanolamine (18:0_20:4)	0.48 (0.22- 1.03	0.08	13	0.978	0.055	0.345	5.740	0.96	1.00
Phosphatidylcholine (18:2_20:4)	0.89 (0.44- 1.82)	0.76	21	0.985	-0.035	0.435	10.350	0.97	0.99
Phosphatidylinositol (16:0_18:1)	0.69 (0.32- 1.46)	0.35	13	0.897	0.005	0.932	8.073	0.89	1.00
Sterol ester (27:1/18:3)	0.73 (0.46- 1.18)	0.22	15	0.974	0.008	0.897	6.789	0.98	1.00
Triacylglycerol (56:4)	2.12 (0.65- 6.88)	0.26	7	0.571	-0.025	0.755	7.464	0.60	0.84
Phosphatidylcholine (O−18:2_20:4)	0.83 (0.29 2.34)	0.73	12	0.802	0.073	0.312	9.457	0.77	0.91

### 3.4 Reverse MR analysis

**Figure 7:**
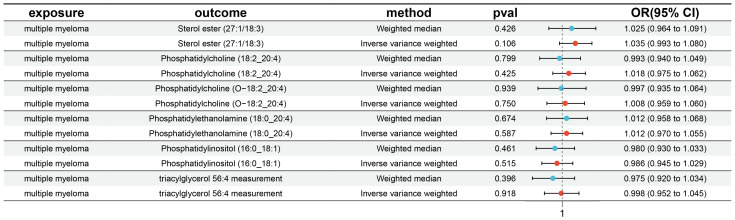
The reverse MR analysis of MM on six plasma lipid.

Furthermore, an inverse MR analysis was conducted on the six significant plasma lipid to investigate potential causal effects of multiple myeloma on these lipid. This analysis adhered to the methodology previously described. Our findings indicated no evidence of reverse causality between multiple myeloma and any of the six significant plasma lipid, as illustrated in **Figure 7.**

## 4 Discussion

In this study, we utilized publicly available genetic datasets on human plasma lipid and multiple myeloma to explore the causal relationships among 179 liposome components and multiple myeloma. Our study marks the first Mendelian randomization analysis to assess the connections between various liposome components and multiple myeloma, revealing a causal link between six liposome components and the disease.

Significantly, we observed a positive association between the serum levels of Triacylglycerol (56:4) and the risk of multiple myeloma, aligning with existing research that has documented alterations in serum triglyceride levels in relation to multiple myeloma. For instance, Małgorzata Kuliszkiewicz-Janus et al. reported a significant elevation in serum triglyceride levels during the active phase of the disease (9). Triglycerides are the main form of fat stored in adipose tissue. Both excessively high and low levels of triglycerides in the blood can be associated with various health issues such as heart disease, diabetes, and metabolic syndrome. Hypertriglyceridemia is a manifestation of hyperlipidemia, and numerous studies have reported its association with an increased risk of multiple myeloma. Furthermore, evidence of high triglyceride levels has been observed in patients with multiple myeloma and benign monoclonal gammopathy (12, 13). The precise mechanisms underlying the relationship between heightened triglyceride levels and multiple myeloma risk remain to be fully elucidated. However, we hypothesize that it may pertain to the energy metabolism of tumor cells. Extensive literature suggests that adipocytes can supply tumor cells with energy through lipids. Given their role in energy storage and supply within adipose tissue and blood, triglycerides are crucial liposomal components that might serve as a readily accessible energy source for lymphoid tumor cells (14). The triglyceride/free fatty acid (TG/FFA) cycle plays a pivotal role in generating signals that regulate a variety of metabolic, physiological, and signaling pathways in the cell (15). This cycle allows for the transient uptake of free fatty acids derived from triglyceride catabolism by cancer cells, facilitating rapid proliferation and the synthesis of cell membrane components. The oxidation of fatty acids within tumor cell mitochondria transforms fatty acids into energy-rich molecules such as NADH, NADPH, FADH2, and ATP, which are essential for the growth and proliferation of cancer cells (16). Panaroni C et al. further demonstrated that multiple myeloma cells could assimilate free fatty acids from triglyceride catabolism through fatty acid transporter proteins, participating in free fatty acid metabolism. This study also revealed a dose-dependent effect of free fatty acids on multiple myeloma cells, with low concentrations promoting cell proliferation and viability, whereas high concentrations adversely affected cell growth and survival (17).

It should be noted that although our study only observed a causal relationship between triglycerides and MM, other manifestations of hyperlipidemia, such as hypercholesterolemia, and even conditions like chylomicron syndrome, are also closely associated with multiple myeloma. Hyperlipidemia is another component of metabolic syndrome and has been reported in patients with MGUS and MM, particularly the immunoglobulin (Ig)-A subtype (18–20). Numerous studies have identified paraproteins as a key factor in the onset of hyperlipidemia associated with MM (21–23). This is thought to be due to the interaction of paraprotein with serum lipoproteins, tissue receptors, and lipoprotein lipase, resulting in decreased lipoprotein clearance. Corsini et al. (23) described a MGUS patient with autoantibodies against the LDL receptor, a finding supported by Nozaki et al. (24), who demonstrated IgA binding at the LDL receptor’s site in an MM patient with autoimmune hyperlipidemia. Additionally, the conversion of IDL to LDL and LDL receptor binding are impaired in MM patients due to immunoglobulin-lipoprotein complexes.

Our research has pinpointed phosphatidylinositol (16:0_18:1) as another liposome component potentially linked to an elevated risk of multiple myeloma. Phosphatidylinositol (PI), a critical constituent of cellular membranes, plays a pivotal role in various biological processes, including intracellular signaling, membrane transport, and cytoskeletal regulation. The involvement of PI and its signal transduction pathways is significant in the pathogenesis of multiple myeloma, with evidence suggesting that its activation is a hallmark of the disease and its inhibition can trigger apoptosis (25). *In vitro* studies lend further support to the relationship between PI and multiple myeloma risk. The application of phosphatidylinositol-3 kinase inhibitors, such as LY294002 and Wortmannin, has been shown to suppress the phosphorylation of key signaling proteins including Akt, FKHRL-1, and p70S6K, thereby markedly reducing the proliferation of multiple myeloma cells (26). Additionally, Tang et al. documented the potent anti-myeloma effects of C96, a phosphatidylinositol-3-kinase (PI3K) inhibitor, in a mouse model of the disease. This study highlighted that C96 curtailed PI3K activation and downregulated the activity of mTOR, p70S6K, and 4E-BP1 in both a time- and concentration-dependent manner, culminating in the apoptosis of multiple myeloma cells (27). These findings indirectly corroborate our observation that phosphatidylinositol is positively correlated with the risk of developing multiple myeloma, underscoring the potential mechanistic link between PI signaling pathways and the progression of this malignancy.

Our analysis identified four liposome components—phosphatidylethanolamine (18:0_20:4), phosphatidylcholine (18:2_20:4), sterol esters (27:1/18:3), and phosphatidylcholine (O-18:2_20:4)—that are potentially associated with a reduced risk of multiple myeloma. Phosphatidylethanolamine (PEA) and phosphatidylcholine are phospholipids integral to cell membranes. While large-scale studies directly linking PEA and phosphatidylcholine to MM risk are lacking, preliminary research suggests a potential role in MM development. For instance, Wilson I. Gonsalves et al. discovered significantly lower serum levels of PEA and phosphatidylcholine in multiple myeloma patients compared to those with monoclonal gammopathy of undetermined significance (MGUS), highlighting potential differences in lipid metabolism between these conditions (28).

Hematological cancer cells, characterized by their rapid proliferation, necessitate ongoing synthesis of phospholipids and sterols for membrane formation. From the perspective of complex lipids, phosphatidylethanolamine and phosphatidylcholine are crucial structural elements of cell membranes. Notably, elevated levels of these phospholipids are associated with a diminished risk of multiple myeloma, implying that such cells might exhibit enhanced utilization of membrane biosynthesis for tumor proliferation. Alternatively, this phenomenon could be attributed to an immune response mechanism. Specifically, natural killer cells and T-lymphocytes, which recognize phosphatidylethanolamine and phosphatidylcholine, may facilitate T-lymphocyte proliferation and augment protein kinase C activity (PKC) in various cell lines (29, 30). Research indicates that elevated levels of phosphatidylethanolamine and phosphatidylcholine can markedly enhance the effects of the immune system through various mechanisms. Initially, these phospholipids interact with membrane-associated signaling pathways, directly influencing the activation and proliferation of T cells (31). The increase in phospholipids not only improves membrane fluidity but also enhances the organization of receptors on the membrane. This optimization facilitates the interaction between cell surface receptors and their ligands, promoting a rapid response and expansion of immune cells (32, 33). Additionally, phosphatidylethanolamine and phosphatidylcholine play a crucial role in the activation of PKC, a key signaling molecule that governs cell proliferation, differentiation, and survival. In T cells specifically, activation of PKC leads to the migration of transcription factors, thus inducing the expression of immune response genes. Consequently, PEA and PC significantly bolster the immune cells defensive capabilities against infections by regulating PKC activity (30). Moreover, research also suggests that phosphatidylethanolamine may induce apoptosis in tumor cells by activating the ferroptosis pathway in multiple myeloma cells. This effect is mediated as oxygenated phosphatidylethanol interacts with Toll-like receptor 2, facilitating the phagocytosis of iron-rich cells (34).

Sterol esters, formed through the esterification of sterols with fatty acids, are vital lipid components in both plants and animals, playing a crucial role in cell membrane composition. These esters significantly influence the structure and fluidity of cell membranes, thereby impacting cellular interactions (35). Given that sterol components of the cell membrane are crucial for signal transduction, a reduction in sterol ester levels may adversely affect the lipid components within the membrane, thereby influencing the activity of cell surface receptors. Such an alteration in receptor activity could disrupt growth factor-dependent signaling pathways, ultimately promoting the proliferation and survival of myeloma cells (36). Additionally, in the bone marrow microenvironment, the interplay among various cell types, such as osteoblasts, myeloid cells, and immune cells, is critical for maintaining its integrity. A reduction in sterol esters could impair these cellular interactions, potentially destabilizing the bone marrow microenvironment and fostering the onset of hematological malignancies (37).

Furthermore, the regulation of the cell cycle may also be influenced by changes in sterol ester levels. Abnormalities in cholesterol metabolism have been linked to the regulation of cell cycle checkpoints, which can lead to the rapid division of malignant cells by affecting the expression and activity of key cyclins (36, 38). Additionally, a reduction in sterol ester levels may trigger an increase in cellular stress responses, such as oxidative stress or endoplasmic reticulum stress; the latter is often caused by improper protein folding and is particularly pronounced in multiple myeloma cells that secrete large amounts of monoclonal proteins. These cellular responses are pivotal in enabling myeloma cells to adapt to harsh microenvironments, thereby enhancing their survival capabilities (39, 40). Moreover, the modulation of signaling pathways by sterol esters plays a crucial role in the activation and proliferation of B lymphocytes, key precursors in multiple myeloma development (41). However, further research is essential to elucidate the specific mechanisms by which low levels of sterol esters might trigger multiple myeloma.

This study boasts several notable strengths. Primarily, it represents the inaugural MR analysis to scrutinize the causal relationship between 179 liposome components and multiple myeloma, marking a pioneering effort in this research domain. Unlike prior studies that merely correlated lipid components with increased risk of multiple myeloma, our MR design inherently mitigates confounding variables, enhancing the reliability of our findings. Additionally, the employment of instrumental variables, combined with a robust sample size and GWAS data, furnishes our study with ample statistical power to ascertain causality, thereby bolstering its credibility. Nevertheless, the study is not without limitations. The dataset primarily encompasses individuals of European descent, and the potential for participant overlap across datasets exists, which might inflate the estimated effects. Furthermore, as is inherent in all MR analyses, our study cannot completely rule out the influence of unobserved pleiotropy, which may skew the results. Ultimately, relying on datasets from a single database can lead to several potential issues, including data redundancy and potential biases. Moving forward, we aim to access additional databases and conduct further analyses. This will allow us to validate and possibly expand our findings, enhancing the external validity and impact of our research.

## 5 Conclusion

In conclusion, our study sheds light on the relationship between lipid and MM, presenting a detailed exploration of the causal links between specific liposomal components and the disease through MR analysis. We identified a positive association between multiple myeloma and the serum levels of triacylglycerol and phosphatidylinositol. Specifically, heightened serum concentrations of triacylglycerol (56:4) and phosphatidylinositol (16:0_18:1) were linked to an elevated risk of developing multiple myeloma. Conversely, increased levels of phosphatidylethanolamine (18:0_20:4), phosphatidylcholine (18:2_20:4), sterol esters (27:1/18:3), and phosphatidylcholine (O-18:2_20:4) were associated with a reduced risk of the disease. This research contributes valuable insights into the clinical relevance of the relationship between lipid and multiple myeloma, enhancing the potential for incorporating liposomal components into the assessment of patients with this condition.

## Data availability statement

The original contributions presented in the study are included in the article/**Supplementary Material**. Further inquiries can be directed to the corresponding author.

## Author contributions

YZ: Conceptualization, Data curation, Formal analysis, Funding acquisition, Investigation, Methodology, Writing – original draft, Writing – review & editing. YL: Conceptualization, Data curation, Formal analysis, Funding acquisition, Writing – original draft. WS: Conceptualization, Data curation, Formal analysis, Writing – original draft, Writing – review & editing. MX: Conceptualization, Data curation, Formal analysis, Investigation, Methodology, Writing – original draft, Writing – review & editing.
