# Corrigendum: Lipid have a direct effect on multiple myeloma: a Mendelian randomization study

**DOI:** 10.3389/fonc.2025.1603501

**Published:** 2025-05-06

**Authors:** Yingbin Zhong, Yanhao Li, Weipeng Sun, Mingfeng Xiao

**Affiliations:** ^1^ Guangzhou University of Chinese Medicine, Guangzhou, China; ^2^ The First Affiliated Hospital of Guangzhou University of Chinese Medicine, Guangzhou, China; ^3^ Guangdong Clinical Research Academy of Chinese Medicine, Guangzhou, China; ^4^ First Clinical Medical College, Guangzhou University of Chinese Medicine, Guangzhou, China; ^5^ College of Traditional Chinese Medicine, Jinan University, Guangzhou, China

**Keywords:** multiple myeloma, lipid, single nucleotide polymorphisms, Mendelian randomization, genome‐wide association study, causal relationship

In the published article, there was an error in the article title. Instead of “Liposomes have a direct effect on multiple myeloma: A Mendelian randomization study”, it should be “Lipid have a direct effect on multiple myeloma: A Mendelian randomization study”.

In the published article, there was an error in the legend for **Figures 2**–**7**, **Table 1** as published. The term “liposomes” is commonly associated with lipid-based drug delivery systems. In our study, we used it to describe the lipid profile in plasma and its potential impact on disease mechanisms, which may cause confusion. The corrected legends appear below.

**Figure 2:**
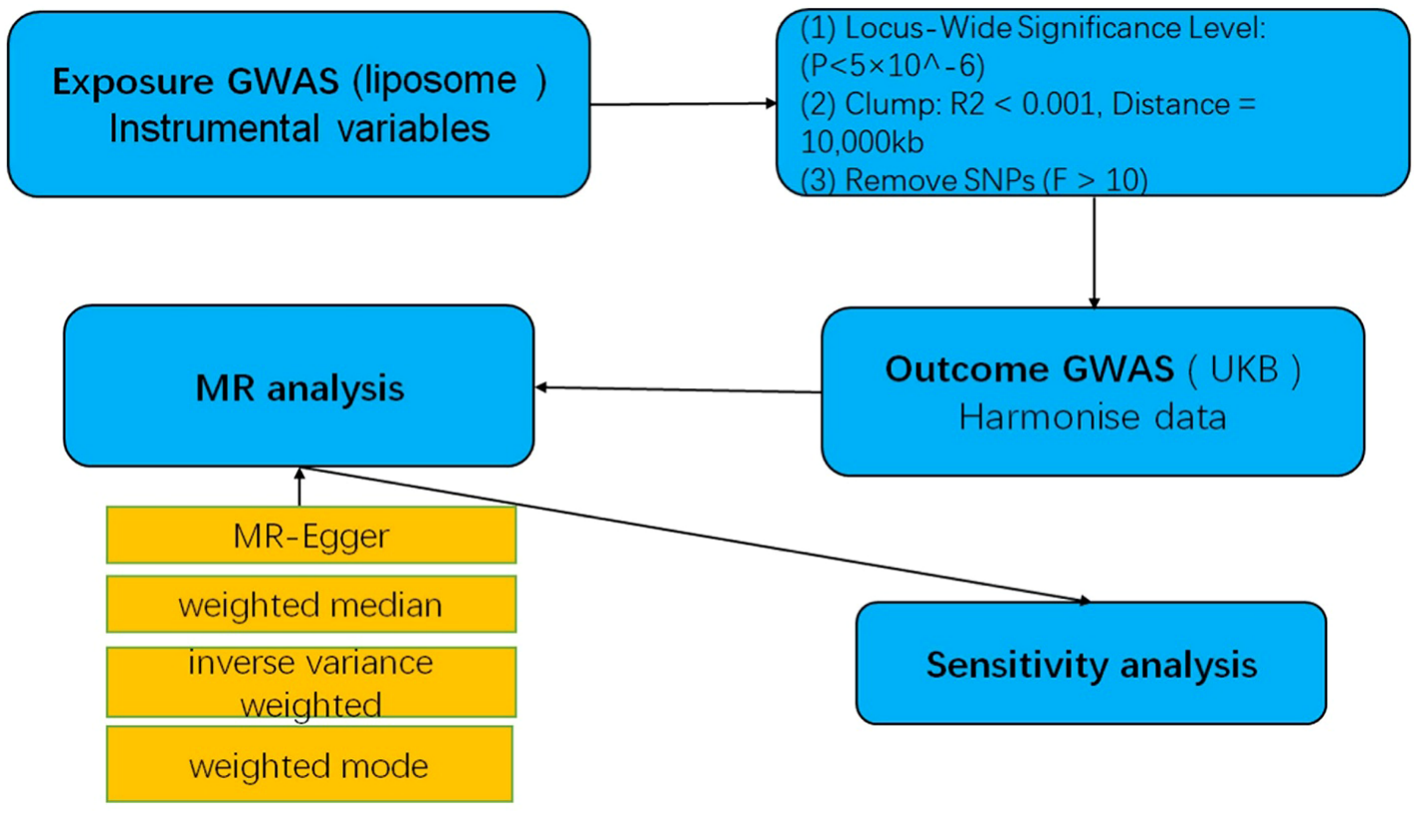
A flow-chart of MR study to explore the causal relationship between lipid and multiple myeloma.

**Figure 3:**
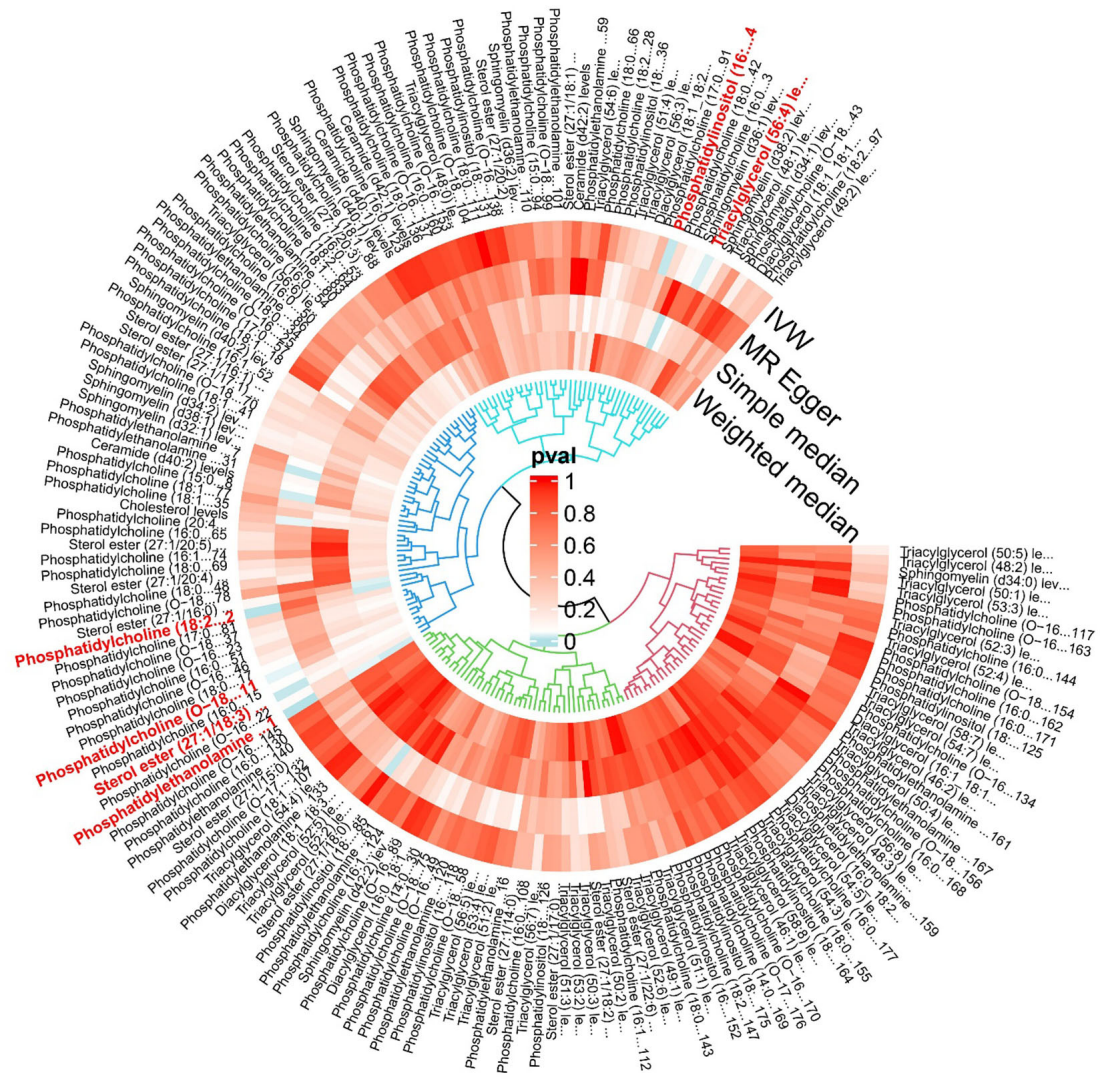
The effect of various lipid on multiple myeloma.

**Figure 4:**
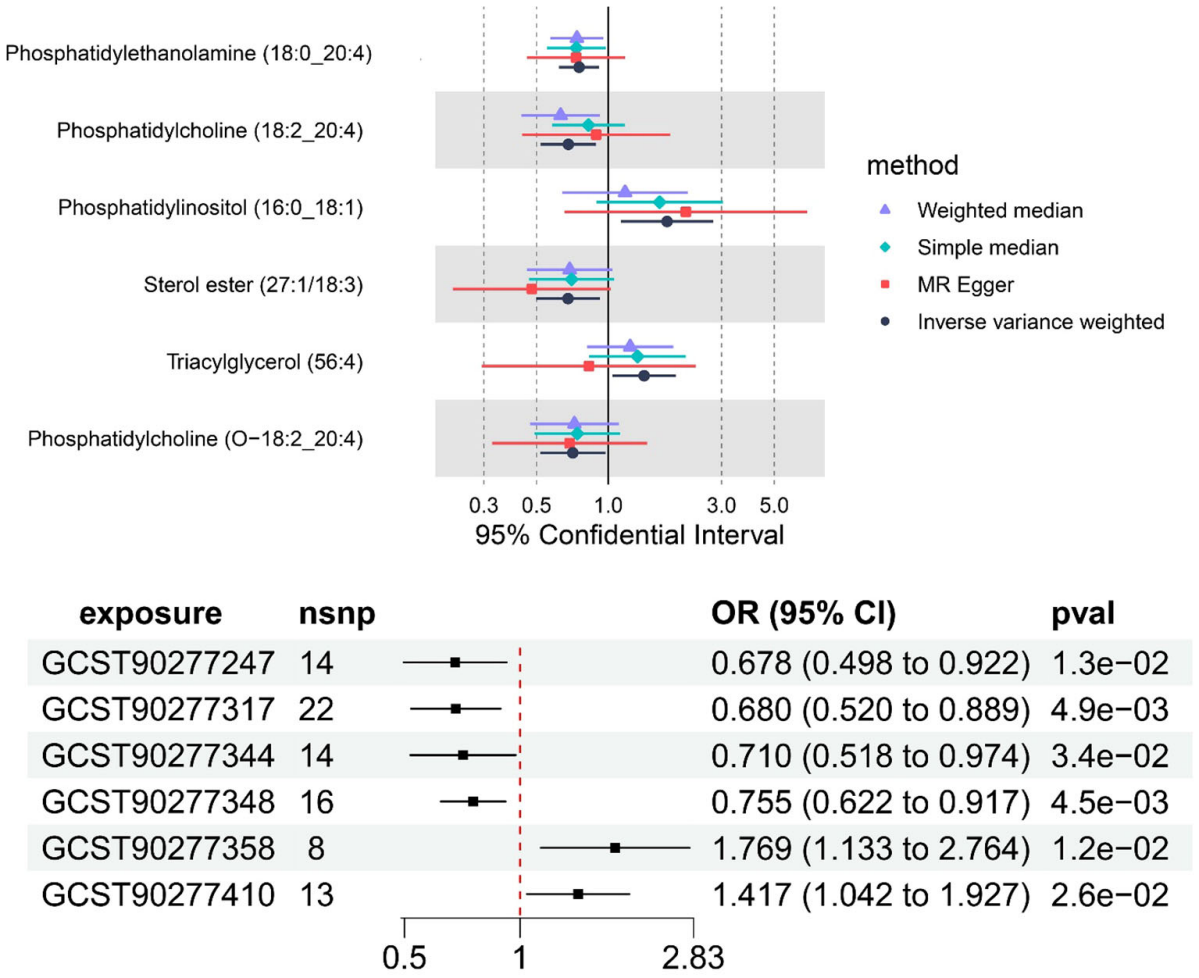
The causal effect of six plasma lipid on MM risk; GCST90277317: Phosphatidylcholine (18:2_20:4) levels; GCST90277247: Sterol ester (27:1/18:3) levels; GCST90277344: Phosphatidylcholine (O-18:2_20:4); GCST90277348: Phosphatidylethanolamine (18:0_20:4); GCST90277358: Phosphatidylinositol (16:0_18:1) levels; GCST90277410: Triacylglycerol (56:4) levels.

**Figure 5:**
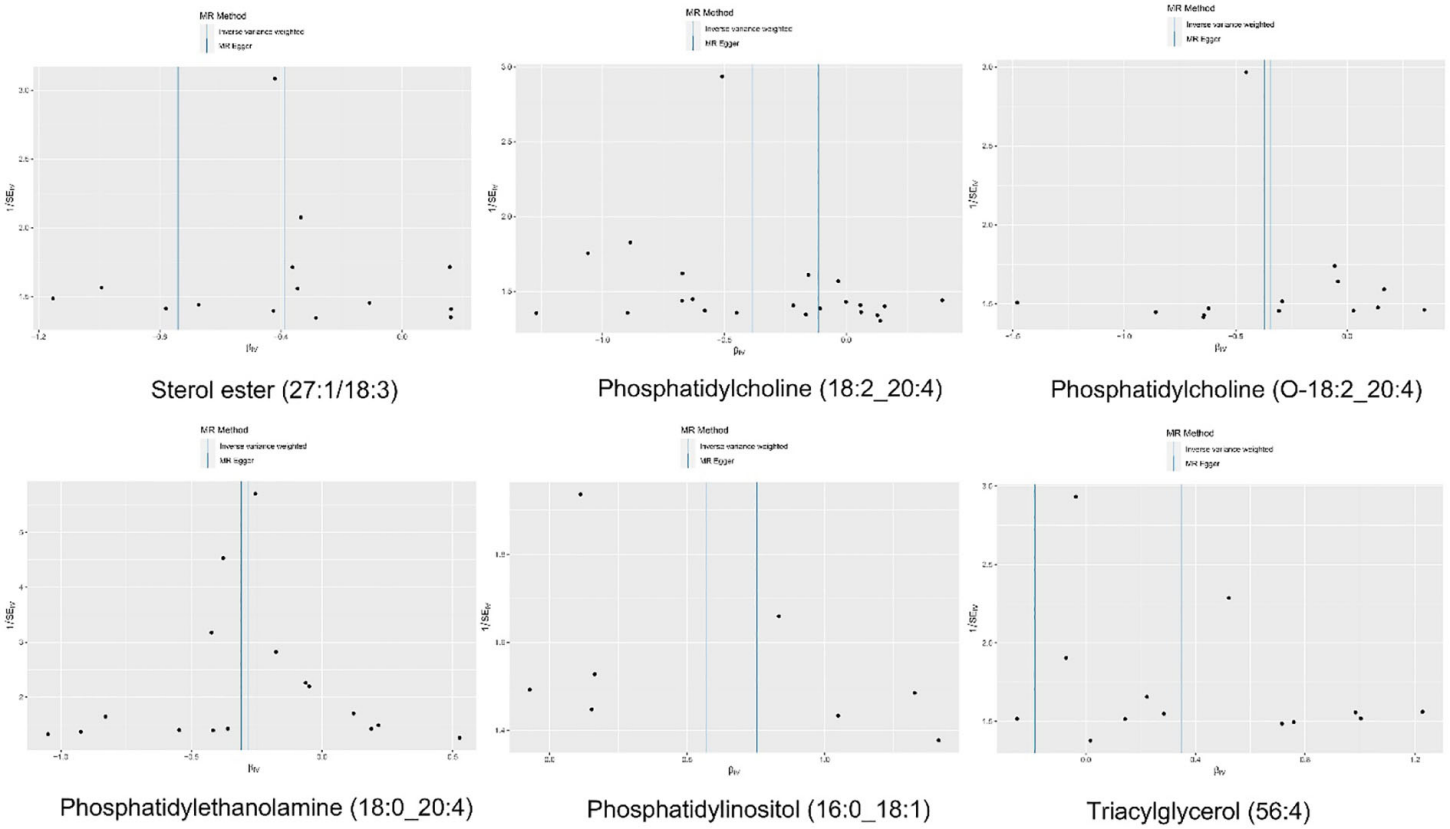
The funnel plot of the causal effect of six plasma lipid on MM risk. It’s almost symmetrical on both sides, which indicated no significant heterogeneity between the effect estimates of the IVs in both the IVW method and MR-Egger method.

**Figure 6:**
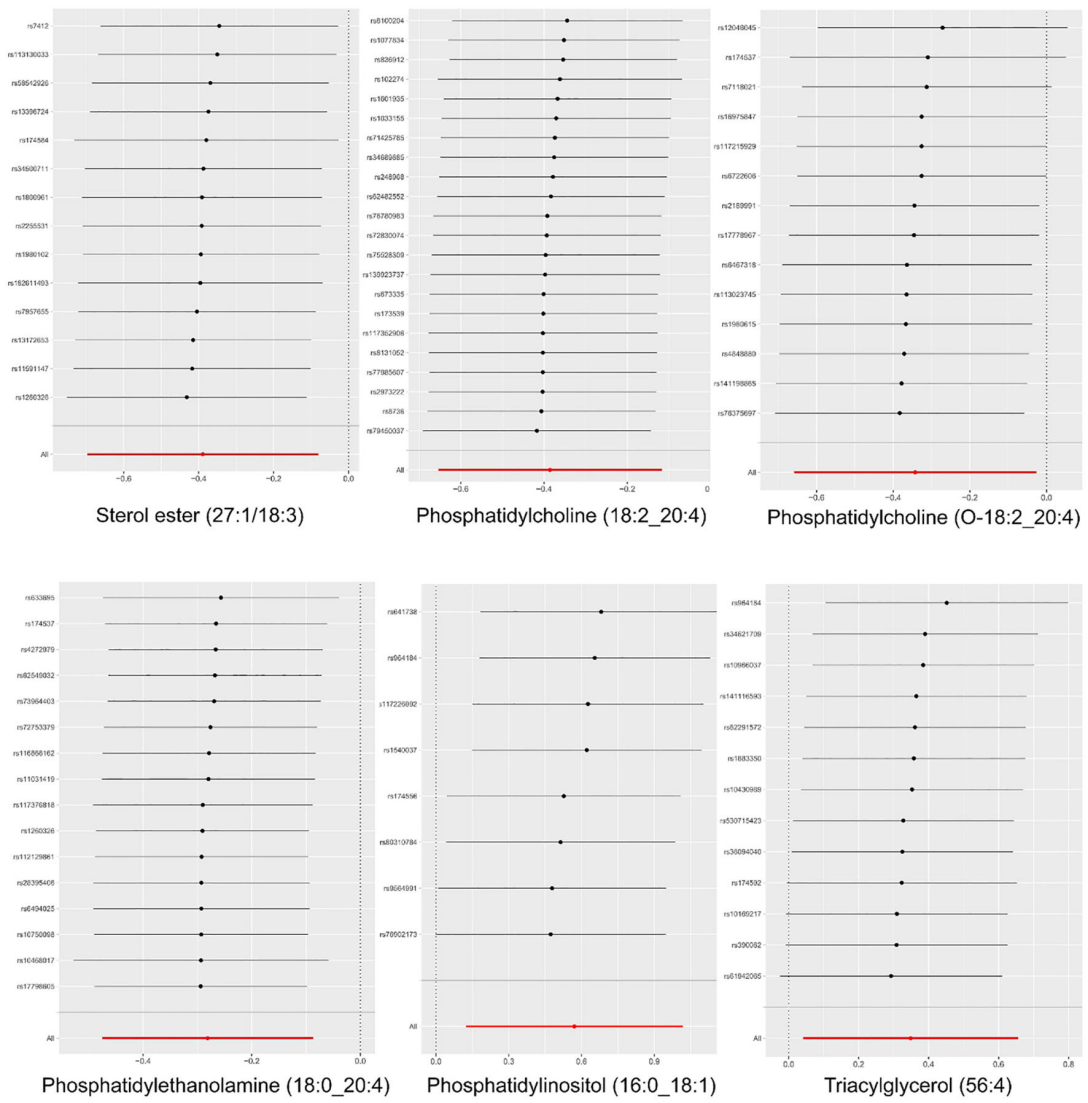
Leave-one-out analysis of the causal association between six lipid and MM. The exclusion of individual SNPs did not result in substantial differences in the combined effect estimates between the remaining SNPs and the overall results.

**Figure 7:**
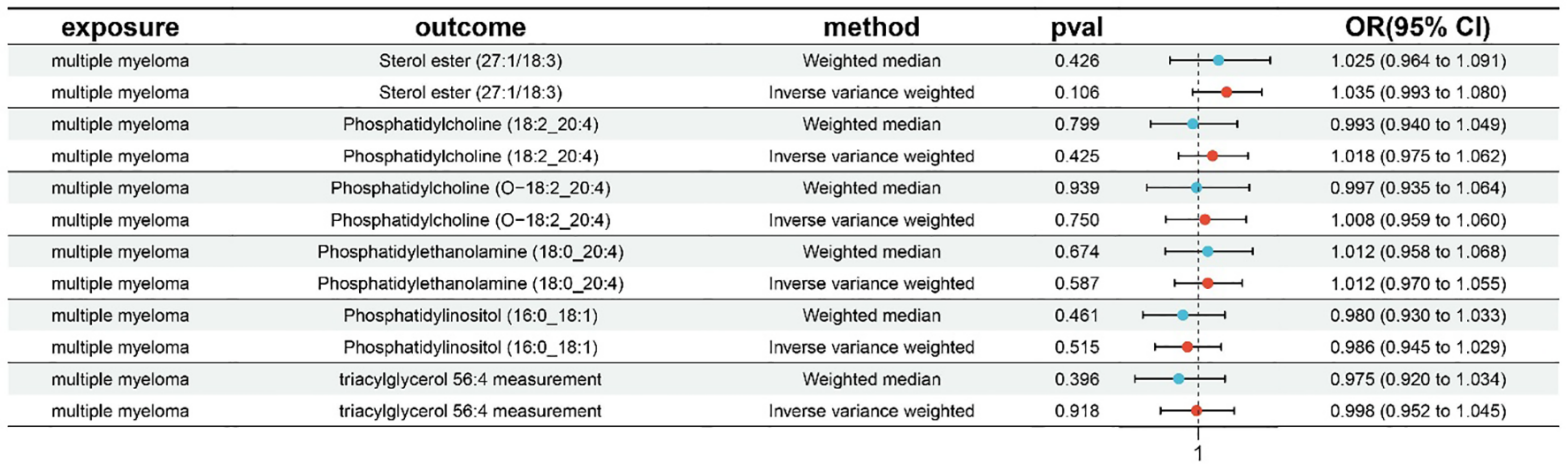
The reverse MR analysis of MM on six plasma lipid.

**Table 1:** Results of multiplicity and sensitivity analyses of six lipid.

Description Lipid	MR-Egger		MR-heterogeneity		MR-pleiotropy		MR-PRESSO_Global		Power
	OR(95%Cl)	p-value	Cochran’s Q	p-value	intercept	p-value	RSS	p-value	
Phosphatidylethanolamine (18:0_20:4)	0.48 (0.22-1.03)	0.08	13	0.978	0.055	0.345	5.740	0.96	1.00
Phosphatidylcholine (18:2_20:4)	0.89 (0.44-1.82)	0.76	21	0.985	-0.035	0.435	10.350	0.97	0.99
Phosphatidylinositol (16:0_18:1)	0.69 (0.32-1.46)	0.35	13	0.897	0.005	0.932	8.073	0.89	1.00
Sterol ester (27:1/18:3)	0.73 (0.46-1.18)	0.22	15	0.974	0.008	0.897	6.789	0.98	1.00
Triacylglycerol (56:4)	2.12 (0.65-6.88)	0.26	7	0.571	-0.025	0.755	7.464	0.60	0.84
Phosphatidylcholine (O−18:2_20:4)	0.83 (0.29-2.34)	0.73	12	0.802	0.073	0.312	9.457	0.77	0.91

“Figure 2 A flow-chart of MR study to explore the causal relationship between lipid and multiple myeloma.”

“Figure 3 The effect of various lipid on multiple myeloma.”

“Figure 4 The causal effect of six plasma lipid on MM risk; GCST90277317: Phosphatidylcholine (18:2_20:4) levels; GCST90277247: Sterol ester (27:1/18:3) levels; GCST90277344: Phosphatidylcholine (O-18:2_20:4); GCST90277348: Phosphatidylethanolamine (18:0_20:4); GCST90277358: Phosphatidylinositol (16:0_18:1) levels; GCST90277410: Triacylglycerol (56:4) levels.”

“Figure 5 The funnel plot of the causal effect of six plasma lipid on MM risk. It’s almost symmetrical on both sides, which indicated no significant heterogeneity between the effect estimates of the IVs in both the IVW method and MR-Egger method.”

“Figure 6 Leave-one-out analysis of the causal association between six lipid and MM.”

“Figure 7 The reverse MR analysis of MM on six plasma lipid.”

“**Table 1** Results of multiplicity and sensitivity analyses of six lipid”.

In the published article, there was an error in **Table 1** as published. The term “liposomes” is commonly associated with lipid-based drug delivery systems. In our study, we used it to describe the lipid profile in plasma and its potential impact on disease mechanisms, which may cause confusion. Therefore, we changed “Liposome” to “Lipid”. The corrected **Table 1** and its caption appear below.

In the published article, the reference for 7-11 which discuss “liposomes” was fully relevant to “lipid” It should be removed.

In the published article, there was an error. The term “liposomes” is commonly associated with lipid-based drug delivery systems. In our study, we used it to describe the lipid profile in plasma and its potential impact on disease mechanisms.

A correction has been made to **Abstract, Introduction, Materials and methods, Results, Discussion, Conclusion**, page 01-09. This word previously stated:

“liposomes”

The corrected sentence appears below:

“lipid”

A correction has been made to **Introduction**. This sentence previously stated:

“Liposomes, artificial bimolecular membranes composed primarily of phospholipids and cholesterol, are endogenous substances inherent to living organisms. Their compatibility with biological tissues and non-immunogenic nature makes them an ideal medium for drug delivery. As a nanoscale drug delivery system, liposomes have garnered interest for their capacity to encapsulate drugs effectively, optimizing their distribution within the body, enhancing targeting precision, and minimizing toxic side effects. In the realm of MM therapy, liposome technology emerges as a promising avenue for developing targeted therapeutic strategies aimed at enhancing treatment efficacy while reducing drug-induced toxicity (10, 11). Recent investigations suggest a significant role for specific human serum liposome components in MM pathogenesis.”

The corrected sentence appears below:

“Recent investigations suggest a significant role for specific human serum lipid components in MM pathogenesis.”

The authors apologize for these errors and state that this does not change the scientific conclusions of the article in any way. The original article has been updated.

